# Magnetic Composites of Dextrin-Based Carbonate Nanosponges and Iron Oxide Nanoparticles with Potential Application in Targeted Drug Delivery

**DOI:** 10.3390/nano12050754

**Published:** 2022-02-24

**Authors:** Fabrizio Caldera, Roberto Nisticò, Giuliana Magnacca, Adrián Matencio, Yousef Khazaei Monfared, Francesco Trotta

**Affiliations:** 1 Department of Chemistry, NIS Centre, University of Torino, Via P. Giuria 7, 10125 Torino, Italy; fabrizio.caldera@unito.it (F.C.); giuliana.magnacca@unito.it (G.M.); adrian.matencioduran@unito.it (A.M.); yousef.khazaeimonfared@unito.it (Y.K.M.); 2 Department of Materials Science, University of Milano-Bicocca, Via R. Cozzi 55, 20125 Milano, Italy

**Keywords:** magnetic nanocomposites, dextrin nanosponges, cyclodextrin, maltodextrin, iron oxide nanoparticles, doxorubicin, targeted drug delivery

## Abstract

Magnetically driven nanosponges with potential application as targeted drug delivery systems were prepared via the addition of magnetite nanoparticles to the synthesis of cyclodextrin and maltodextrin polymers crosslinked with 1,1′-carbonyldiimidazole. The magnetic nanoparticles were obtained separately via a coprecipitation mechanism involving inorganic iron salts in an alkaline environment. Four composite nanosponges were prepared by varying the content of magnetic nanoparticles (5 wt% and 10 wt%) in the cyclodextrin- and maltodextrin-based polymer matrix. The magnetic nanosponges were then characterised by FTIR, TGA, XRD, FESEM, and HRTEM analysis. The magnetic properties of the nanosponges were investigated via magnetisation curves collected at RT. Finally, the magnetic nanosponges were loaded with doxorubicin and tested as a drug delivery system. The nanosponges exhibited a loading capacity of approximately 3 wt%. Doxorubicin was released by the loaded nanosponges with sustained kinetics over a prolonged period of time.

## 1. Introduction

Cyclodextrins (CDs) and polysaccharide-based materials have been extensively investigated as innovative drug delivery systems. CDs are cyclic oligosaccharides with a poorly hydrophilic central cavity in which nonpolar organic molecules of compatible size can be trapped, forming an inclusion complex [1]. Polysaccharides—and in particular maltodextrins, with high content of amylose—have been proposed as more economical alternatives to CDs, although the binding constants of the inclusion complexes formed by the amylose helix are usually weaker compared to CDs [2,3]. The polymerisation of CDs and maltodextrins with linking agents leads mainly to crosslinked polymer structures, often referred to as “nanosponges” (NSs). NSs can form complexes with a wider series of guest molecules than the native dextrins due to the presence of hydrophilic pores among the dextrin molecules. Because of their outstanding complexation properties, ability to release encapsulated drugs with controlled kinetics, and intrinsic nontoxicity, CDs and maltodextrin NSs are among the most promising excipients for drug delivery formulations. Nevertheless, the release of drugs from NSs is neither site-specific nor “on demand” [4,5].

To overcome such limitations, stimuli-responsive cyclodextrin NSs capable of enhancing drug release upon triggering have recently been developed. Trotta et al. synthesised a redox-responsive NS by introducing disulphide links in the polymer structure of a pyromellitic β-CD NS, and then used it to encapsulate doxorubicin (Doxo)—an anticancer drug. As observed, the release of Doxo was magnified in the presence of oxidised glutathione—a reducing agent found at high concentrations inside chemoresistant tumour cells [6]. Momin et al. used the same NS to encapsulate erlotinib; the complex was then administered to BALB/c mice that had been injected with lung carcinoma epithelial cells. The mice treated with the erlotinib-loaded redox-responsive NS showed reduced side effects to healthy tissues compared to the mice treated with the free drug [7]. A later study also investigated the pH-dependent behaviour of this NS; this feature, which derives from the carboxyl groups of the pyromellitic units, was exploited to regulate the release of plant hormone analogues (strigolactones) with chemotherapeutic activity [8]. Another example of a pH-sensitive NS was developed by Fontana et al. by functionalising a network polymer of calixarene and β-CD with carboxyl and amine groups; the ability of the NS to release drugs at different pH values was then evaluated using a tetracycline antibiotic as a model drug [9].

As regards maltodextrin-based and, more generally, polysaccharide-based stimuli-responsive polymers, several examples have been described in the literature. The list includes temperature-responsive, pH-responsive, ionic-strength-responsive, light-responsive, and solvent-responsive polymers [10].

The next step towards the development of smart drug delivery systems involves the preparation of nanocarriers that are able to reach specific target sites and then accumulate over time, so that the release of drugs will be limited to the diseased tissue. To this end, two main approaches are currently being studied in the field of nanomedicine: The first concerns the decoration of nanocarrier particles with specific biologically active molecules that can recognize the receptors of a target cell and selectively bind to them [11,12]. The second strategy consists of the preparation of magnetic drug delivery systems that can be actively driven to the site of interest by the application and movement of an external magnetic field [13,14,15].

Over the years, numerous magnetic materials have been proposed as scaffolds for the design of drug carriers; among these, magnetite is one of the most used. Magnetite is a ferrimagnetic material, usually indicated as Fe_3_O_4_, resulting from the combination of two iron oxide structures (i.e., FeO and Fe_2_O_3_). When the particle size of magnetite is smaller than 20 nm, it exhibits superparamagnetic behaviour, with high magnetic susceptibility [16]. In recent years, magnetite nanoparticles have been used as a contrast agent in magnetic resonance imaging [17,18,19], as well as in clinical trials for thermotherapy of tumours, as they can generate heat upon external application of an alternating magnetic field [20,21]. In addition, several drug shuttles based on magnetite nanoparticles have been obtained either by functionalising the surface of the nanoparticles with organic molecules—able to form complexes or conjugates with drugs [22,23,24]—or by coating the nanoparticles with layers of macromolecules on which drugs can be adsorbed [25,26,27]. In most cases, polymeric coatings interact weakly with the nanoparticle surface, resulting in low stability over time, whereas chemical modification of the nanoparticle surface by covalent bonding usually requires complex multistep procedures. CDs have also been used to decorate magnetite nanoparticles [28,29,30,31]. However, the thin layer of CD macromolecules around the nanoparticles and the ability to host drugs only in the internal cavity of CDs, with a release profile that depends mainly on the drug–CD binding constant, may limit the overall performance of the nanocarrier. NSs have already been combined with magnetic nanoparticles in a study by Salazar et al., and then used for environmental applications [32]; however, as the magnetic NSs were prepared by physically mixing a pre-synthesised NS with magnetite nanoparticles, it is likely that the magnetic nanoparticles were distributed mostly on the surface of the NS particles, and interacted loosely with it. Therefore, the long-term stability of the magnetic properties of such material may be an issue of concern.

In this paper, we describe the preparation of magnetic nanocomposites for targeted drug delivery, in which magnetite/maghemite nanoparticles are embedded in a dextrin-based nanosponge. This material intrinsically combines the magnetic features of iron oxide nanoparticles [33,34,35] with the controlled drug release kinetics and encapsulation capacity of CD-based NSs. To achieve a permanent and homogeneous physical entrapment of the magnetic nanoparticles within the polymer matrix, the nanoparticles were produced via a coprecipitation mechanism by regulating the molar ratio of Fe(III)/Fe(II) to form magnetite nanostructures. Moreover, iron oxide nanoparticles thus obtained were dispersed in the CD solution before the addition of the crosslinker. In the same way, two NSs with different loads of magnetic nanoparticles were synthesised by replacing β-CD with a highly water-soluble maltodextrin, with an amylose content of approx. 40 wt%, and marketed by Roquette Frères under the name Linecaps (LC). A characterisation study of the synthesised NSs was performed using FTIR, TGA, XRD, FESEM, and HRTEM analysis. Meanwhile, the magnetic properties of the bare magnetite/maghemite nanoparticles and the magnetic NSs were investigated via magnetisation curves collected at RT.

Finally, the ability of the magnetic NSs to efficiently encapsulate an anticancer drug molecule and release it with controlled kinetics was assessed in vitro, using Doxo as a model drug.

## 2. Materials and Methods

β-CD and LC were kindly gifted by Roquette Frères (Lille, France); they were both dried to constant weight in an oven at 80 °C before use. The other chemicals mentioned in this study were purchased from Sigma-Aldrich (Milan, Italy) and used as received, with the exception of N,N-dimethylformamide (DMF), which was treated with CaH_2_ to remove the water content, and then filtered before use.

### 2.1. Synthesis of Magnetite Nanoparticles

Magnetic nanoparticles were prepared following a modified procedure already reported in the literature [33,36]. In detail, approximately 3.70 g of FeCl_3_ and approximately 4.17 g of FeSO_4_·7H_2_O (thus maintaining the molar ratio of Fe(III)/Fe(II) = 1.5) were dissolved in 100 mL of deionised water and heated up to 90 °C. At this target temperature, a previously prepared solution of 28–30% ammonium hydroxide (10 mL) was added. The final mixture was mechanically stirred at 90 °C for 30 min and then cooled down to room temperature (RT). The obtained precipitates were magnetically separated using a commercial neodymium magnet, and then purified by washing several times with deionised water, deposited in a glass Petri dish, and oven-dried at 80 °C overnight. The obtained materials were stored dried at RT before use in closed containers; under these conditions they show long-term stability [37].

### 2.2. Synthesis of Magnetic Nanosponges

**Figure 1:**
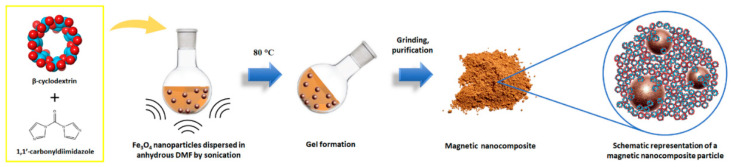
Preparation steps of a β-CD-based magnetic nanosponge.

β-CD-based magnetic NSs bearing a content of 5 and 10 wt% of Fe_3_O_4_ (values referred to the theoretical weight of the purified NSs) were synthesised following the procedure depicted in Figure 1, and named β5% and β10%. Additionally, two NSs were prepared using the same method, but replacing β-CD with an equal amount of a linear pea starch derivative—Linecaps (LC)—and named LC5% and LC10%. More specifically, a selected amount of iron oxide nanoparticles (the exact quantities are listed in Table 1) was finely dispersed in 20 mL of anhydrous DMF by intense sonication. Then, anhydrous β-CD (or LC) and 1,1′-carbonyldiimidazole (CDI) were added to the mixture, and the temperature was increased up to 80 °C. The amount of CDI used for the synthesis corresponded to a CDI/β-CD molar ratio of 8, and resulted in a CDI/glucopyranose molar ratio of approximately 1.14, for all of the NSs (LC has a wide distribution of molecular weight; therefore, the exact CDI/LC molar ratio cannot be defined). Heating and sonication were prolonged until a rigid NS gel was formed. After grinding, the NSs were purified by washing with deionised water, Soxhlet extraction with ethanol and, finally, recovered in the form of dry powders, consisting of iron oxide nanoparticles dispersed in a carbonate-crosslinked dextrin network.

### 2.3. Preparation of Fe_3_O_4_-Decorated NS

An Fe_3_O_4_-decorated NS was prepared by physically mixing magnetite nanoparticles with a β-CD carbonate NS, following the method described by Salazar et al. [32], with minor modifications. More specifically, a mass of 200 mg of NS was stirred in 100 mL of a dispersion of Fe_3_O_4_ nanoparticles. A few hours later, the stirring was stopped, and the Fe_3_O_4_-decorated NS was recovered by centrifugation and, finally, freeze-dried.

The Fe_3_O_4_-decorated NS structure and its stability over time were investigated in comparison with the synthesised nanocomposite materials.

### 2.4. Fourier-Transform Infrared Spectroscopy (FTIR)

The FTIR spectra of the magnetic NSs were recorded with a PerkinElmer 100 FTIR (Waltham, MA, USA) using an attenuated total reflectance (ATR) accessory. All of the samples were scanned in the 4000–650 cm^−1^ range at a resolution of 4 cm^−1^, collecting 8 scans per spectrum.

### 2.5. Thermogravimetric Analysis (TGA)

The thermal stability of the synthesised magnetic nanocomposites was investigated using a TA Instruments Q500 TGA (New Castle, DE, USA). The analyses were performed on approximately 10 mg of sample, placed in an alumina pan, with a heating ramp of 10 °C/min from RT to 700 °C in air. The content of magnetic nanoparticles in the nanocomposite samples was calculated, taking into account the weight loss of the magnetic nanoparticles that were analysed via the same method.

### 2.6. CHNS Analysis

The elemental composition of the organic fraction of the magnetic nanocomposites was assessed with a Thermo Fisher CHNS-O analyser Flash EA 1112 series (Waltham, MA, USA). Approximately 2.5 mg of each sample was introduced to a tin crucible and mixed with an equal amount of catalyst (V_2_O_5_). The percentage contents of N, C, H, and S were determined from the area of the chromatographic peaks, using a multiple-point external calibration curve of 2–3 mg of 2,5-Bis(5-ter-butyl-benzoxazol-2-yl) thiophene (BBOT). All of the samples were analysed in triplicate.

### 2.7. X-ray Powder Diffraction Studies (XRD)

The crystalline structure of the magnetic nanocomposites was investigated with a Malvern Panalytical X’Pert diffractometer (Worcestershire, UK), using Cu Kα1 as a source of radiation. Data were collected over an angular range from 20 to 70° 2θ, using a step size of 0.017° 2θ and a time per step of 59.69 s.

### 2.8. FESEM Analysis

Field-emission scanning electron microscopy (FESEM) images were collected using an FESEM TESCAN S9000G (Brno, Czech Republic) with an FEG Schottky source. Before observation, the samples were coated with a 5 nm thick Cr layer.

### 2.9. HRTEM Analysis

High-resolution transmission electron microscopy (HRTEM) micrographs were acquired on a JEOL 3010-UHR HRTEM microscope (Musashino Akishima, Japan) combined with a 2000 × 2000 pixel Gatan US1000 CCD camera (Pleasanton, CA, USA). The microscope operated at 200 kV, with a resolution of 0.12 nm.

### 2.10. Magnetisation Curves

The magnetisation curves were recorded with a Lake Shore 7404 vibrating sample magnetometer (Universidad Nacional de La Plata, Argentina). The hysteresis loop of all samples was collected at RT, and the magnetic field was cycled between −20,000 and 20,000 Oe.

### 2.11. Stability Study

The stability over time of the nanocomposites’ structure was studied in comparison with the Fe_3_O_4_-decorated NS by monitoring the content of carbon over prolonged washing cycles. A quantity of 100 mg each of the nanocomposite β10% and the Fe_3_O_4_-decorated NS were thoroughly stirred in two separate vials containing 10 mL of deionised water. At fixed intervals (i.e., 24, 32, and 48 h), a strong magnet was placed under each vial to attract and quickly precipitate the magnetic materials. Then, the supernatant was removed, and a few mg of precipitate was recovered and freeze-dried for CHNS analysis. Finally, the supernatant was replaced with 10 mL of fresh, deionised water, and the dispersions were stirred again.

### 2.12. Loading of Doxo in Magnetic Dextrin-Based Nanosponges

A concentrated Doxo solution was prepared by stirring 250 mg of Doxo in 23 mL of ultrapure water at RT (Doxo solubility in water = 10 mg/mL). After 24 h, the solution was filtered (0.45 µm). Then, 100 mg of NS was added to 5 mL of Doxo solution and stirred for 24 h. After centrifugation (10 min at 3000× *g* rpm), the loaded NS was recovered and freeze-dried.

### 2.13. Quantification of the Content of Doxo

The amount of Doxo loaded in the NSs was determined by extracting 10 mg of NS in 1 mL of water–ACN (75:25) solution. After centrifugation, the supernatant was completely removed, filtered (0.45 µm) for HPLC analysis, and replaced with 1 mL of fresh solution (HPLC method: column C18 Luna 150 mm × 4.6 mm × 5 µm, mobile phase 1% acetic acid–acetonitrile 75–25 *v*/*v*, λ detector 254 nm, flow 1 mL/min, 8 min total run time, elution time ca. 2.5 min, external calibration with 1, 2.5, 5, 7.5, 10, 25, 50, 75 and 100 μg/mL Doxo). The extraction was repeated eight times. The loading capacity and encapsulation efficiency of the NSs were calculated as follows:

(1) $$Loading\;capacity\;\left(\%\right)=\frac{mass\;of\;loaded\;Doxo\;\left(mg\right)}{mass\;of\;NS\;\left(mg\right)}*100\;$$

(2) $$Encapsulation\;efficiency\;\left(\%\right)=\frac{mass\;of\;loaded\;Doxo\;\left(mg\right)}{mass\;of\;used\;Doxo\;\left(mg\right)}*100\;$$

### 2.14. Release Studies

Release simulation in physiological buffer was performed at 37 °C in phosphate buffer solution (PBS) at pH 7.4. Specifically, 10 mg of NS was added to 2 mL of PBS and stirred. After a defined time, the dispersions were centrifuged. Then, 1 mL of supernatant was recovered, filtered (0.45 µm) for HPLC analysis, and replaced with 1 mL of fresh PBS. The amount of Doxo released at each time was determined using the HPLC method described above. Finally, the cumulative release profiles were fitted using a number of mathematical models (i.e., pseudo-first-order kinetics, pseudo-second-order kinetics, Higuchi simplified model, Hixson–Crowell kinetics, Korsmeyer–Peppas model, Weibull model, and Peppas–Sahlin model) to determine which model had the highest correlation with the experimental data.

## 3. Results and Discussion

Four magnetic NSs—namely, β5%, β10%, LC5%, and LC10%—were successfully prepared by adding different amounts (5 and 10 wt%) of magnetic nanoparticles during the synthesis of β-CD and LC carbonate NSs. The mass balance values of the synthesis reaction of the NSs, calculated as the ratio of the purified product to the theoretical weight of the NS, were found to be in the range of 80–90%.

### 3.1. Physicochemical Characterisation

**Figure 2:**
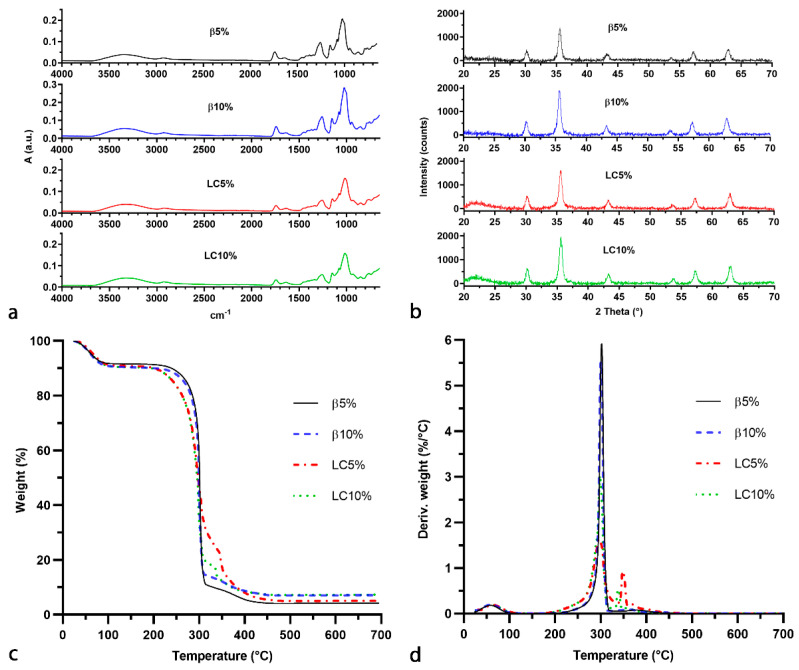
(**a**) FTIR-ATR, (**b**) XRD, (**c**) TGA, and (**d**) DTG analysis of β5%, β10%, LC5%, and LC10%.

FTIR–ATR analysis (Figure 2a) performed on the four magnetic NSs allowed us to confirm the expected composition of the polymer matrix. The stretching vibrations of the O-H, C-H, and C-O bonds of the dextrin units appeared between 3600–3000 cm^−1^, 2950–2850 cm^−1^, and 1250–1000 cm^−1^, respectively. The signal at 1630 cm^−1^ can be ascribed to the bending mode of the OH groups of the dextrin molecules, whereas the presence of the crosslinking units is confirmed by the peak at 1750 cm^−1^, which derives from the stretching vibrations of the C=O bonds.

The exact content of magnetic nanoparticles in the four composites was determined by TGA analysis performed in air (Figure 2c,d). The oxidative degradation of β5%, β10%, LC5%, and LC10% led to a final residue at 700 °C, corresponding to contents of nanoparticles of 4.96, 8.35, 5.98, and 8.58 wt%, respectively. Additionally, the initial part of the thermograms shows a weight loss before 100 °C of approximately 10 wt%, which is due to the release of the moisture adsorbed by the hydrophilic dextrin molecules. Overall, the magnetic composites based on LC were observed to be stable up to 175 °C, whilst the β-CD NSs started degrading at 225 °C, because of the higher thermal stability of CDs with respect to maltodextrins.

CHNS analysis (Table 2) confirmed the presence of a major organic fraction in the nanocomposite samples. A small percentage of nitrogen (between 0.3 and 0.5%), due to either residual imidazole or DMF, was detected in all of the samples. However, the absence of a peak around 153 °C (the boiling point of DMF) in the first derivative of the thermograms (Figure 2c,d) indicates that nitrogen belongs mainly to the imidazole groups. It is noteworthy that imidazole groups covalently bonded to the polymer structure might be exploited as reactive leaving groups for further chemical functionalisation of the NSs. The C/H weight ratio of the samples (i.e., 6.8–7.0) was significantly lower than the theoretical ratio of βNS-CDI(1:8) (i.e., 8.5) and imidazole (i.e., 8.9); this was probably due to the presence of water, as demonstrated by TGA analysis.

The identification of the iron oxide phase in the nanocomposites was assessed by XRD analysis (Figure 2b). The signals at 2θ = 30.1° (220), 35.4° (311), 43.0° (400), 53.9° (422), 57.2° (511), and 62.6° (440) correspond to the main reflections of the magnetite/maghemite phase (card numbers 00-019-0629 and 00-039-1346, ICCD Database). No relevant reflections were expected from either β-CD or LC NSs, since their XRD pattern presents only a few negligible broad bands at 2θ < 30°, indicating an amorphous polymer structure with only short-range order.

**Figure 3:**
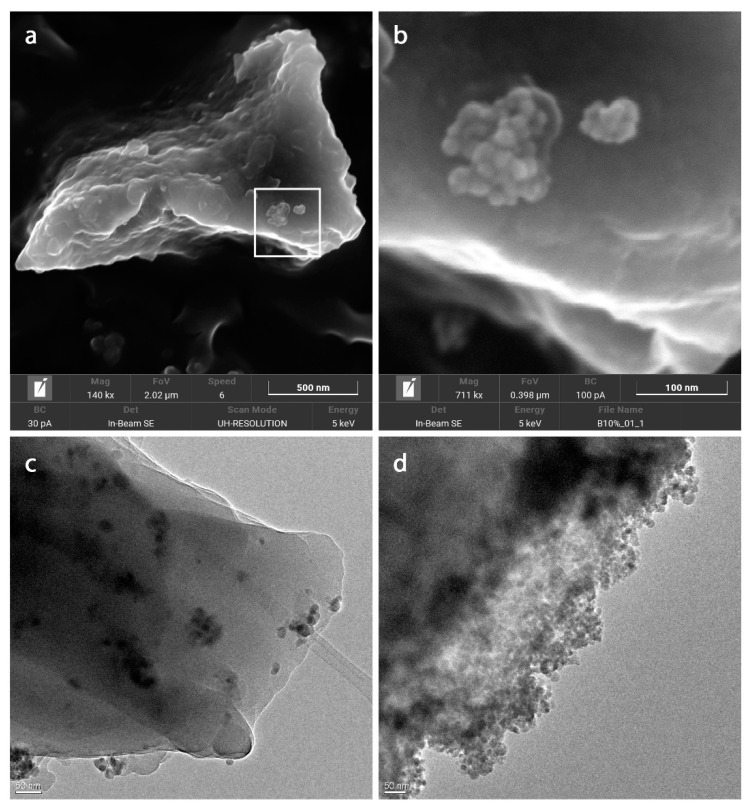
(**a**) FESEM image of β10% (140 kx); (**b**) higher magnification of the square drawn in (**a**) (771 kx); (**c**) HRTEM micrograph of β10% (30 kx); (**d**) the Fe_3_O_4_-decorated NS (25 kx).

The morphology and particle size of the nanocomposite materials were examined via FESEM. Figure 3a shows a micrometric particle of the nanocomposite β10% bearing a small agglomerate of Fe_3_O_4_ nanoparticles on its surface (further magnified in Figure 3b). The chemical composition of the iron oxide nanoparticles was confirmed by EDX analysis. In general, the synthesised nanocomposites exhibited particles of irregular morphology, with very few iron oxide nanoparticles (∼10 nm diameter) exposed on the surface.

HRTEM analysis of the nanocomposites was then performed in order to better evaluate the distribution of the magnetic nanoparticles within the polymer network. An Fe_3_O_4_-decorated NS was observed under the same conditions in comparison to our nanocomposites. Figure 3c shows the presence of small aggregates of magnetic nanoparticles homogeneously dispersed in the NS matrix. Meanwhile, in the case of the Fe_3_O_4_-decorated NS, the magnetic nanoparticles were thickly packed on the surface of the NS particles (Figure 3d).

**Figure 4:**
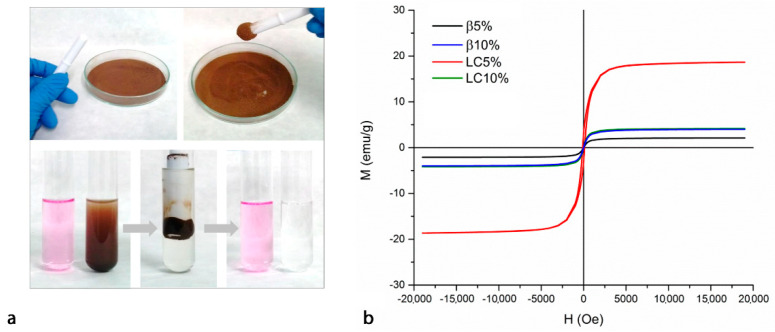
(**a**) Removal of phenolphthalein from an aqueous solution by treatment with magnetic NS β10%. (**b**) Magnetisation curves evaluating LC5% (red line), LC10% (green line), β5% (black line), and β10% (blue line).

A simple qualitative test to evaluate the magnetic properties and encapsulation capacity of the synthesised NSs is presented in Figure 4a. Even the application of a relatively weak magnetic force, created using a magnetic stir bar, was enough to attract and move the NS particles. The addition of 100 mg of β10% to 5 mL of a diluted phenolphthalein solution enabled adsorption of the entire amount of colourant. After the magnetically driven removal of the NS, the solution appeared colourless.

The magnetic properties of the four NSs were more accurately evaluated by means of magnetisation curves collected at RT (magnetisation curves’ profiles are reported in Figure 4b, whereas numerical values are summarised in Table 3). For comparison, numerical values associated with bare magnetite were taken from a previous study [37]. As suggested by the very narrow hysteresis loop, all samples clearly exhibited superparamagnetic behaviour, although the hysteresis was more pronounced in the case of LC5% [16,34]. According to the literature [34,38], saturation magnetisation (Ms) is the maximum magnetic moment induced by an externally applied magnetic field, magnetic remanence (Mr) is the residual magnetisation at zero external magnetic field (H = 0), whereas intrinsic coercivity (Hic) is the reverse field required to bring the magnetisation M to zero. In general, results indicate that all magnetic NSs show magnetic properties very similar between one another, with the only exception being represented by LC5%.

With respect to bare magnetite, all magnetic NSs show remarkably lower saturation magnetisation (i.e., bare magnetite’s Ms value is 64 emu/g), with values of 19 emu/g (LC5%), 4 emu/g (LC10%), 2 emu/g (β5%), and 4 emu/g (β10%). The difference in terms of saturation magnetisation is mainly due to the presence of the non-magnetic polymeric coatings surrounding the iron oxide nanoparticles, which cause a quenching of the surface magnetic moment [34,37]. Interestingly, Ms values of βCD-based NSs are consistent with the polymeric content (i.e., the higher the polymeric shell, the lower the Ms values), whereas this trend is the opposite in the case of LC-based NSs.

Concerning the other two parameters—magnetic remanence (Mr) and intrinsic coercivity (Hic)—LC10% and both βCD-based NSs have low Mr (approximately 0.1 emu/g, whereas bare magnetite is 1.0 emu/g) and low Hic (approximately 11–14 Oe, whereas bare magnetite is 10 Oe). Conversely, LC5% shows higher Mr (approximately 3.1 emu/g) and a remarkably higher Hic value (120 Oe). High values of Mr and Hic indicate the capability of a magnetic material to retain a memory of its magnetic history, thus behaving as a permanent magnet.

**Figure 5:**
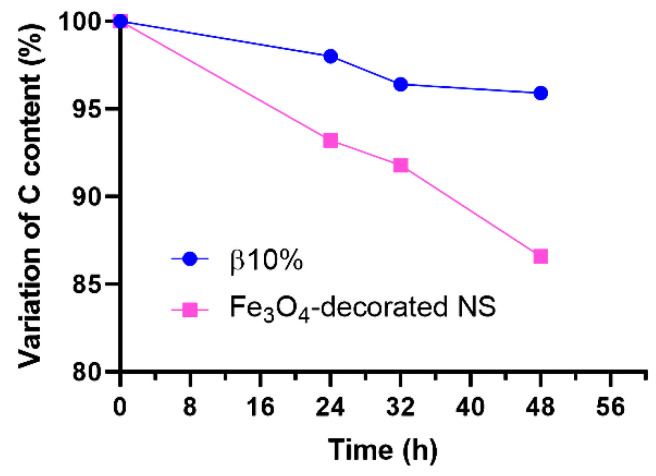
Variation in the content of carbon in β10% and the Fe_3_O_4_-decorated NS over a prolonged washing cycle.

The stability of the nanocomposite physical structure under repeated washing cycles was investigated by stirring the sample β10% in water for a prolonged time and then checking the content of carbon that remained in the magnetic fraction. For comparison, the experiment was performed on the Fe_3_O_4_-decorated NS as well. The variation in the content of C, as detected by CHNS analysis, is presented in Figure 5.

While the content of C in the magnetic fraction of β10% decreased by only 4% over 48 h, the Fe_3_O_4_-decorated NS showed a loss of almost 14% of the initial amount of C, thus indicating that the magnetic nanoparticles were separating from the organic NS, which will inevitably result in lower encapsulation efficiency of the magnetic fraction of the material. This outcome, along with both the FESEM and HRTEM analyses, confirms the hypothesis that the magnetic nanoparticles of the synthesised nanocomposites are mainly entrapped in the bulk of the polymer particles, rather than loosely attached to the surface.

### 3.2. Encapsulation of Doxorubicin and Release Study

Four Doxo-loaded magnetic NSs were prepared by stirring β5%, β10%, LC5%, and LC10% in a saturated Doxo solution. After filtration and freeze-drying, the NSs were extracted with a water–ACN (75/25) solution. For the quantification of Doxo, an external calibration curve (y = 0.386x − 0.4911) was successfully obtained in the concentration range 1–100 µg/mL, with a correlation coefficient r^2^ = 0.9993.

**Figure 6:**
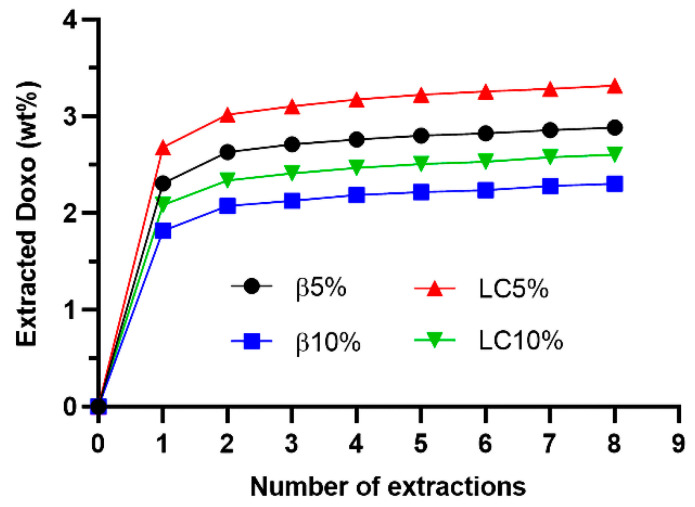
Extraction kinetics of Doxo from Doxo-loaded NSs: LC5%, LC10%, β5%, and β10%.

Figure 6 shows the extraction kinetics of Doxo from the loaded NSs. As expected, higher amounts of Doxo were detected in the NSs with a lower percentage of magnetic nanoparticles and lower amount of crosslinker, meaning a higher content of complexing dextrin. Most of the loaded Doxo was removed from the NSs during the first extraction. Such fast release was probably due to the fraction of Doxo adsorbed on the surface of NS particles and weakly interacting with the NSs’ inner structure. Afterwards, the amount of extracted Doxo increased slowly, according to linear kinetics. The loading capacity and encapsulation efficiency values, calculated according to Equations (1) and (2), are listed in Table 4.

Since the amount of extracted Doxo did not reach a plateau, even after eight extractions (Figure 6), the values presented in Table 4 might be slightly underestimated. This indicates a good interaction between the loaded drug and the encapsulating polymer structure. Overall, the NSs were able to encapsulate approximately 2.5–3.5 wt% of Doxo. This is in accordance with what was previously reported in the literature with respect to the encapsulation of Doxo in carbonate β-CD NSs [39]. NSs with a lower content of magnetic nanoparticles (i.e., 5 wt%) exhibited slightly higher loading capacity and encapsulation efficiency.

**Figure 7:**
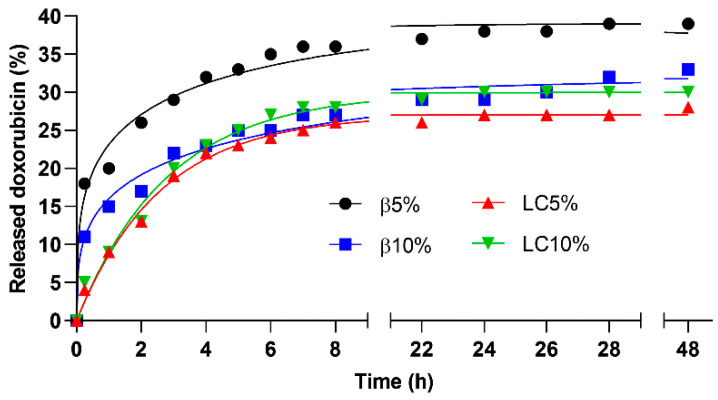
Cumulative release of Doxo from the Doxo-loaded nanocomposites.

The release simulation in physiological buffer of Doxo from the magnetic NSs is presented in Figure 7. The release kinetics were faster during the first 8 h, when approximately 30% of the loaded Doxo was released. Afterwards, less than 5% of the drug was released in the 8–48 h interval. Sustained release kinetics could be observed for all four NSs during the first 8 h, with a fast initial release in the case of β5% and β10%, of approximately 18% and 11% of loaded Doxo within the first 15 min, respectively (Figure 7). This fast release was probably due to the fraction of Doxo loosely adsorbed on the NS particles. The release kinetics exhibited by LC5% and LC10% followed a pseudo-first-order model, with r^2^ of 0.9933 and 0.9911, respectively. Meanwhile, the release profiles of β5% and β10% were best fitted by the Peppas–Sahlin model (r^2^ of 0.9830 and 0.9819, respectively), indicating that in the βCD-based nanocomposites the release rate might be affected by two contributions (diffusional and relaxational) [40].

## 4. Conclusions

Finding ways to release drugs with controlled kinetics and only to target tissues is a key step in the development of new generations of smart drug delivery systems able to enhance the therapeutic efficacy of pharmacological treatments with decreased administration dosage and reduced side effects.

Inspired by this challenge, we successfully synthesised four magnetic NSs by homogeneously dispersing different amounts of Fe_3_O_4_ nanoparticles (5 and 10 wt%) in the polymer network of β-CD and LC-based NSs. As the Fe_3_O_4_ nanoparticles were introduced to the monomer solutions before starting the crosslinking reaction, the polymeric network of the NSs was built around the magnetic nanoparticles, thus providing intimate and stable physical entrapment of the nanoparticles within the polymer structure and, therefore, durable magnetic properties.

The physicochemical characterisation confirmed the formation of a polymeric network of dextrin units and carbonate bridges encapsulating iron oxide magnetic nanoparticles. The application of the magnetic nanocomposites as a drug delivery system was assessed in vitro using Doxo as a model drug. The release of Doxo was sustained during the first 8 h. After 48 h, approximately 30% of the total amount of loaded drug was released by the polymers.

Overall, the findings of this study indicate that the developed magnetic NSs are a promising prototype for a new family of smart drug delivery systems with potential application in targeted therapies.

## Figures and Tables

**Table 1:** Quantities of chemicals used for the synthesis of the magnetic NSs.

Sample	DMF (mL)	Fe_3_O_4_ Nanoparticles (g)	β-CD (g)	LC (g)	CDI (g)
β5%	20	0.031	3.333	-	3.810
β10%	20	0.061	3.333	-	3.810
LC5%	20	0.031	-	3.333	3.810
LC10%	20	0.061	-	3.333	3.810

**Table 2:** CHNS analysis of the four magnetic nanocomposites.

Sample	N (%)	C (%)	H (%)	S (%)
β5%	0.33	37.65	5.52	0.00
β10%	0.52	37.93	5.39	0.00
LC5%	0.29	37.88	5.49	0.00
LC10%	0.30	37.16	5.36	0.00

**Table 3:** Magnetic properties registered at RT for all samples, namely, LC5%, LC10%, β5%, and β10%.

Samples	Saturation Magnetisation, Ms (emu/g of Material)	Saturation Magnetisation, Ms (emu/g of Iron Oxide)	Magnetic Remanence, Mr (emu/g of Material)	Intrinsic Coercivity, Hic (Oe)	Ref.
Magnetite	-	64	1.0	10	[37]
β5%	2	43	0.1	12	Present study
β10%	4	48	<0.1	14	Present study
LC5%	19	312	3.1	120	Present study
LC10%	4	48	0.1	11	Present study

**Table 4:** Absorption and encapsulation of Doxo. Loading capacity and encapsulation efficiency of the tested magnetic NSs.

Doxo-Loaded NSs	Loading Capacity (%)	Encapsulation Efficiency (%)
β5%	2.88	5.30
β10%	2.31	4.25
LC5%	3.32	6.11
LC10%	2.60	4.78

## Data Availability

Not applicable.
